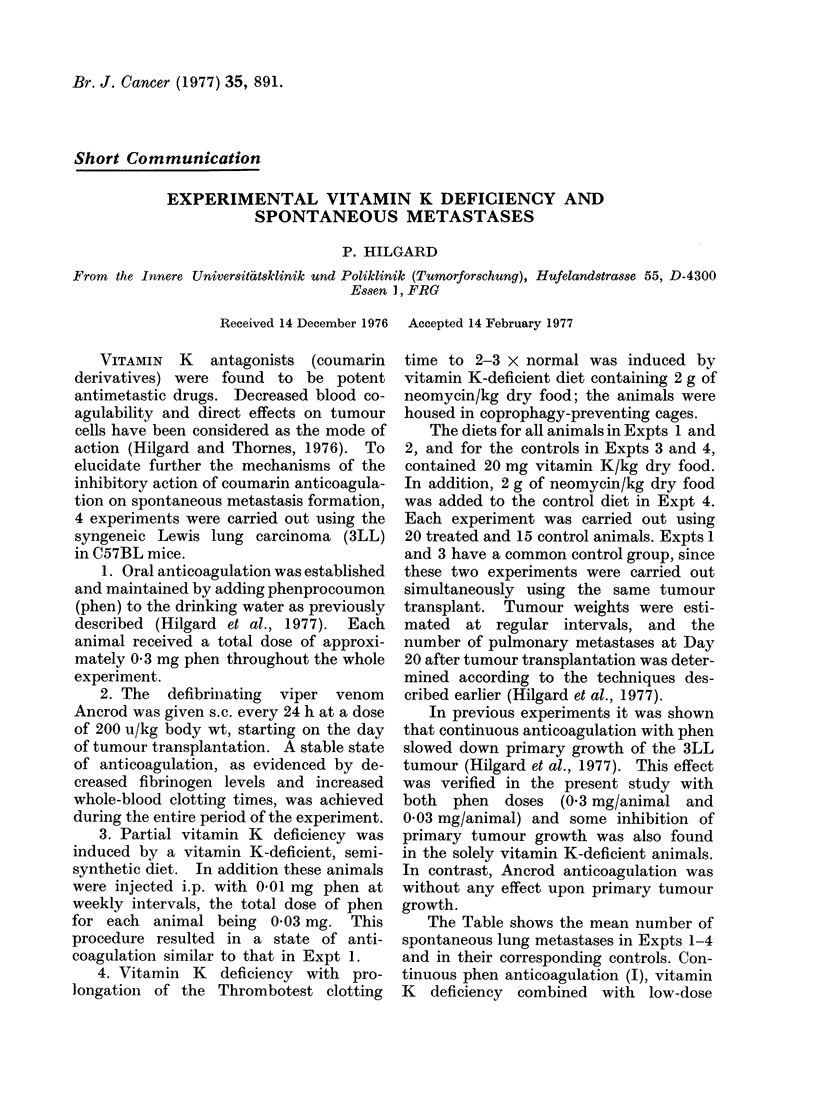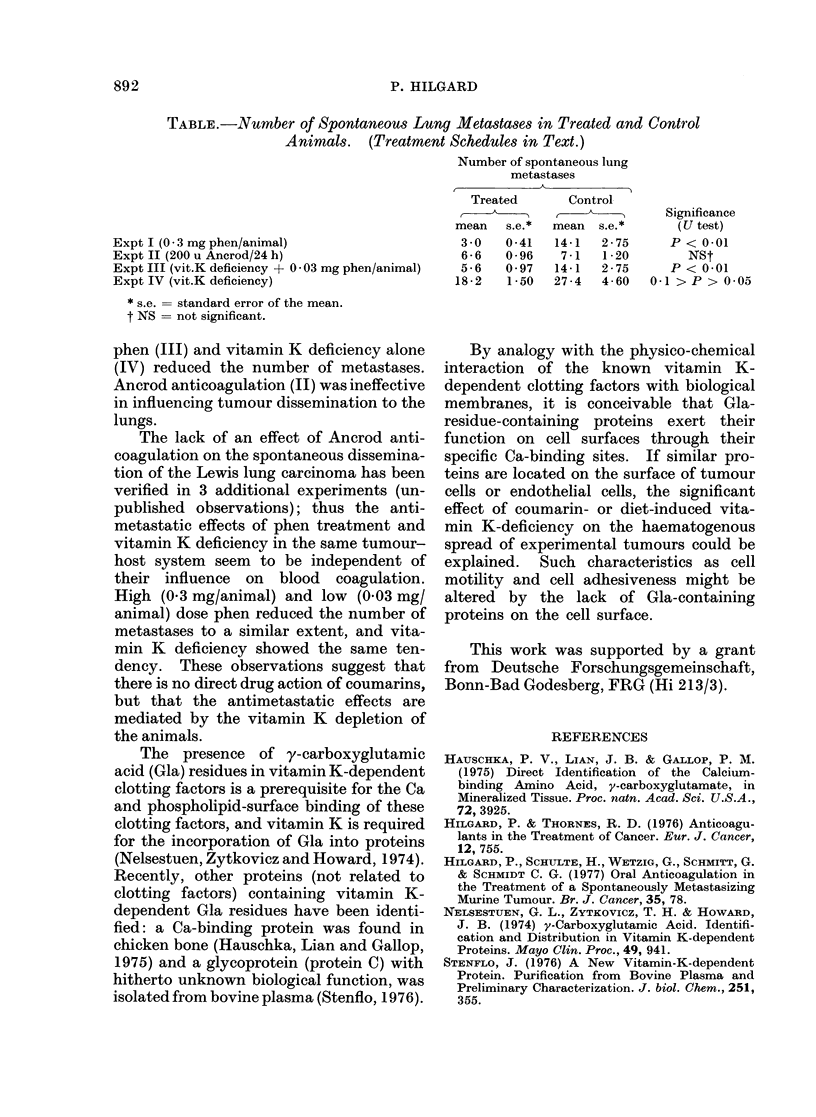# Experimental vitamin K deficiency and spontaneous metastases.

**DOI:** 10.1038/bjc.1977.135

**Published:** 1977-06

**Authors:** P. Hilgard


*Br. J. Cancer* (1977) **35**, 891.

***Short Communication***

### EXPERIMENTAL VITAMIN K DEFICIENCY AND SPONTANEOUS METASTASES

P. HILGARD

*From the Innere Universitätsklinik und Poliklinik (Tumorforschung), Hufelandstrasse* 55, *D*-4300 *Essen* 1, *FRG*

Received 14 December 1976 Accepted 14 February 1977

VITAMIN K antagonists (coumarin derivatives) were found to be potent antimetastic drugs. Decreased blood coagulability and direct effects on tumour cells have been considered as the mode of action (Hilgard and Thornes, 1976). To elucidate further the mechanisms of the inhibitory action of coumarin anticoagulation on spontaneous metastasis formation, 4 experiments were carried out using the syngeneic Lewis lung carcinoma (3LL) in C57BL mice.

1. Oral anticoagulation was established and maintained by adding phenprocoumon (phen) to the drinking water as previously described (Hilgard *et al.*, 1977). Each animal received a total dose of approximately 0·3 mg phen throughout the whole experiment.

2. The defibrinating viper venom Ancrod was given s.c. every 24 h at a dose of 200 u/kg body wt, starting on the day of tumour transplantation. A stable state of anticoagulation, as evidenced by decreased fibrinogen levels and increased whole-blood clotting times, was achieved during the entire period of the experiment.

3. Partial vitamin K deficiency was induced by a vitamin K-deficient, semisynthetic diet. In addition these animals were injected i.p. with 0·01 mg phen at weekly intervals, the total dose of phen for each animal being 0·03 mg. This procedure resulted in a state of anticoagulation similar to that in Expt 1.

4. Vitamin K deficiency with prolongation of the Thrombotest clotting time to 2–3 × normal was induced by vitamin K-deficient diet containing 2 g of neomycin/kg dry food; the animals were housed in coprophagy-preventing cages.

The diets for all animals in Expts 1 and 2, and for the controls in Expts 3 and 4, contained 20 mg vitamin K/kg dry food. In addition, 2 g of neomycin/kg dry food was added to the control diet in Expt 4. Each experiment was carried out using 20 treated and 15 control animals. Expts 1 and 3 have a common control group, since these two experiments were carried out simultaneously using the same tumour transplant. Tumour weights were estimated at regular intervals, and the number of pulmonary metastases at Day 20 after tumour transplantation was determined according to the techniques described earlier (Hilgard *et al.*, 1977).

In previous experiments it was shown that continuous anticoagulation with phen slowed down primary growth of the 3LL tumour (Hilgard *et al.*, 1977). This effect was verified in the present study with both phen doses (0·3 mg/animal and 0·03 mg/animal) and some inhibition of primary tumour growth was also found in the solely vitamin K-deficient animals. In contrast, Ancrod anticoagulation was without any effect upon primary tumour growth.

The Table shows the mean number of spontaneous lung metastases in Expts 1–4 and in their corresponding controls. Continuous phen anticoagulation (I), vitamin K deficiency combined with low-dose

TABLE.—*Number of Spontaneous Lung Metastases in Treated and Control Animals.* (*Treatment Schedules in Text.*)

| | Number of spontaneous lung metastases | | | | |
|---|---|---|---|---|---|
| | Treated | | Control | | Significance (*U* test) |
| | mean | s.e.* | mean | s.e.* | |
| Expt I (0·3 mg phen/animal) | 3·0 | 0·41 | 14·1 | 2·75 | $P < 0{\cdot}01$ |
| Expt II (200 u Ancrod/24 h) | 6·6 | 0·96 | 7·1 | 1·20 | NS† |
| Expt III (vit.K deficiency + 0·03 mg phen/animal) | 5·6 | 0·97 | 14·1 | 2·75 | $P < 0{\cdot}01$ |
| Expt IV (vit.K deficiency) | 18·2 | 1·50 | 27·4 | 4·60 | $0{\cdot}1 > P > 0{\cdot}05$ |

* s.e. = standard error of the mean.
† NS = not significant.

phen (III) and vitamin K deficiency alone (IV) reduced the number of metastases. Ancrod anticoagulation (II) was ineffective in influencing tumour dissemination to the lungs.

The lack of an effect of Ancrod anticoagulation on the spontaneous dissemination of the Lewis lung carcinoma has been verified in 3 additional experiments (unpublished observations); thus the antimetastatic effects of phen treatment and vitamin K deficiency in the same tumour–host system seem to be independent of their influence on blood coagulation. High (0·3 mg/animal) and low (0·03 mg/animal) dose phen reduced the number of metastases to a similar extent, and vitamin K deficiency showed the same tendency. These observations suggest that there is no direct drug action of coumarins, but that the antimetastatic effects are mediated by the vitamin K depletion of the animals.

The presence of $\gamma$-carboxyglutamic acid (Gla) residues in vitamin K-dependent clotting factors is a prerequisite for the Ca and phospholipid-surface binding of these clotting factors, and vitamin K is required for the incorporation of Gla into proteins (Nelsestuen, Zytkovicz and Howard, 1974). Recently, other proteins (not related to clotting factors) containing vitamin K-dependent Gla residues have been identified: a Ca-binding protein was found in chicken bone (Hauschka, Lian and Gallop, 1975) and a glycoprotein (protein C) with hitherto unknown biological function, was isolated from bovine plasma (Stenflo, 1976).

By analogy with the physico-chemical interaction of the known vitamin K-dependent clotting factors with biological membranes, it is conceivable that Gla-residue-containing proteins exert their function on cell surfaces through their specific Ca-binding sites. If similar proteins are located on the surface of tumour cells or endothelial cells, the significant effect of coumarin- or diet-induced vitamin K-deficiency on the haematogenous spread of experimental tumours could be explained. Such characteristics as cell motility and cell adhesiveness might be altered by the lack of Gla-containing proteins on the cell surface.

This work was supported by a grant from Deutsche Forschungsgemeinschaft, Bonn-Bad Godesberg, FRG (Hi 213/3).

### REFERENCES

HAUSCHKA, P. V., LIAN, J. B. & GALLOP, P. M. (1975) Direct Identification of the Calcium-binding Amino Acid, $\gamma$-carboxyglutamate, in Mineralized Tissue. *Proc. natn. Acad. Sci. U.S.A.*, **72**, 3925.

HILGARD, P. & THORNES, R. D. (1976) Anticoagulants in the Treatment of Cancer. *Eur. J. Cancer*, **12**, 755.

HILGARD, P., SCHULTE, H., WETZIG, G., SCHMITT, G. & SCHMIDT C. G. (1977) Oral Anticoagulation in the Treatment of a Spontaneously Metastasizing Murine Tumour. *Br. J. Cancer*, **35**, 78.

NELSESTUEN, G. L., ZYTKOVICZ, T. H. & HOWARD, J. B. (1974) $\gamma$-Carboxyglutamic Acid. Identification and Distribution in Vitamin K-dependent Proteins. *Mayo Clin. Proc.*, **49**, 941.

STENFLO, J. (1976) A New Vitamin-K-dependent Protein. Purification from Bovine Plasma and Preliminary Characterization. *J. biol. Chem.*, **251**, 355.